# Low Circulating Protein C Levels Are Associated with Lower Leg Ulcers in Patients with Diabetes

**DOI:** 10.1155/2013/719570

**Published:** 2013-01-02

**Authors:** K. Whitmont, G. Fulcher, I. Reid, M. Xue, K. McKelvey, Y. Xie, M. Aboud, C. Ward, M. M. Smith, A. Cooper, L. March, C. J. Jackson

**Affiliations:** ^1^Department of Dermatology, The University of Sydney, St Leonards, NSW 2065, Australia; ^2^Sutton Arthritis Research Laboratory, Institute of Bone and Joint Research, Kolling Institute of Medical Research, Royal North Shore Hospital, The University of Sydney, St Leonards, NSW 2065, Australia; ^3^Department of Endocrinology, The University of Sydney, St Leonards, NSW 2065, Australia; ^4^Department of Haematology, Kolling Institute of Medical Research, Royal North Shore Hospital, The University of Sydney, St Leonards, NSW 2065, Australia; ^5^Raymond Purves Research Laboratories, Institute of Bone and Joint Research, Kolling Institute of Medical Research, Royal North Shore Hospital, The University of Sydney, St Leonards, NSW 2065, Australia

## Abstract

Activated protein C (APC) promotes angiogenesis and reepithelialisation and accelerates healing of diabetic ulcers. The aim of this study was to determine the relationship between the incidence of lower leg ulcers and plasma levels of APC's precursor, protein C (PC), in diabetic patients. Patients with diabetes who had a lower leg ulcer(s) for >6 months (*n* = 36) were compared with age-, type of diabetes-, and sex-matched subjects with diabetes but without an ulcer (*n* = 36, controls). Total PC was assessed using a routine PC colorimetric assay. There was a significantly (*P* < 0.001) lower level of plasma PC in patients with ulcers (103.3 ± 22.7, mean ± SD) compared with control (127.1 ± 34.0) subjects, when corrected for age and matched for gender and type of diabetes. Ulcer type (neuropathic, ischaemic, or mixed) was not a significant covariate for plasma PC levels (*P* = 0.35). There was no correlation between PC levels and gender, type of diabetes, HbA_1c_, or C-reactive protein in either group. In summary, decreased circulating PC levels are associated with, and may predispose to, lower leg ulceration in patients with diabetes.

## 1. Introduction 

Activated protein C (APC) is a plasma protease derived from its precursor, protein C (PC), which circulates in plasma at 3–5 *µ*g/mL. APC was originally described as an anticoagulant but has recently been found to exert potent cytoprotective properties including the inhibition of inflammation and apoptosis and maintenance of the endothelial and epithelial barriers [1–4]. APC exerts its cytoprotective effect through its receptor, endothelial protein C receptor (EPCR), which binds to both PC and APC with high affinity [5]. A soluble form of EPCR (sEPCR), circulating in normal human plasma [6], has similar affinity for binding PC as that of intact membrane- bound EPCR. 

In humans, recombinant APC reduces the mortality rate in severe sepsis [7], and we have recently shown its potential application in the healing of chronic wounds [8, 9]. Interestingly, biopsies taken immediately adjacent to chronic wounds in patients with diabetes exhibit very low total PC (PC plus APC) levels compared to normal skin [9]. In animal models, APC is neuroprotective after stroke onset [10], protects diabetic nephropathy [11], significantly inhibits the development of lung fibrosis in bleomycin-induced lung injury [12], reduces intestinal injury in necrotizing enterocolitis [13], and accelerates healing in streptozotocin-induced diabetic rats [14]. In vitro, APC modulates keratinocyte and endothelial function towards a phenotype necessary to promote wound healing by enhancing reepithelialisation and angiogenesis [15–17]. Notably, total PC expression in skin surrounding lower leg ulcers in diabetic patients is lower than normal skin [9]. 

Taken together, these findings triggered our hypothesis that low total PC levels may predispose to lower leg ulcers in diabetes. The aim of the present study was to determine if an association exists between circulating levels of total PC and lower leg ulcers in patients with diabetes. 

## 2. Methods 

A total of 72 outpatients with diabetes mellitus participated. This study was approved by the Northern Sydney Health Human Research Ethics Committee, and written informed consent was obtained from each subject. The diagnosis of either type 1 or 2 diabetes mellitus was made according to the criteria of the American Diabetes Association [18]. Thirty-six patients had at least one lower leg ulcer, and these patients were matched for age, gender, and type of diabetes with 36 patients with diabetes with no history of previous or current lower leg ulcer. Ulcer types were classified as neuropathic (*n* = 14), ischemic (*n* = 10), mixed neuropathic/ischemic (*n* = 11) or venous (*n* = 1). Peripheral ischemia was determined by absence of both dorsalis pedis and posterior tibial pulses on clinical palpation. Peripheral neuropathy was assessed by clinical insensitivity to a 10-gram monofilament. All ulcers were located at or below the malleolus, except the venous ulcer which was located on the lower leg. Our control group consisted of matched patients with diabetes because patients with type 1 or type 2 diabetes have altered levels of circulating PC levels compared to normals [19, 20]. Patients on warfarin or any anticoagulant therapy were excluded from the study. 

Blood sampling was carried out in all subjects from an antecubital vein, and plasma was separated. All assays were performed in a routine diagnostic laboratory. Total PC was assessed using the Stachrom PC colorimetric assay after activation of plasma PC with Agkistrodon contortrix venom (Diagnostica Stago, Asniers, France). Protein S and fibrinogen were measured by an immunoturbidimetric and clot-based test, respectively, performed on a fully automated coagulation analyser (STAR by Diagnostica Stago). The prothrombin time (PT), activated partial thromboplastin time (APTT), and International Normalised Ratio (INR) were also performed on the STAR analyser. Soluble (s)EPCR was measured by ELISA (R & D Systems, Minneapolis, USA). 

Data was tested for normality and, if not normally distributed, transformed to a normal distribution before modelling by linear regression using Stata 11.0. Age was included in the regression as a confounding covariate. Differences in proportions were by Fisher's Exact tests. To quantify any significant associations within the data, pairwise Pearson correlation coefficients were calculated. 

## 3. Results

**Figure 1:**
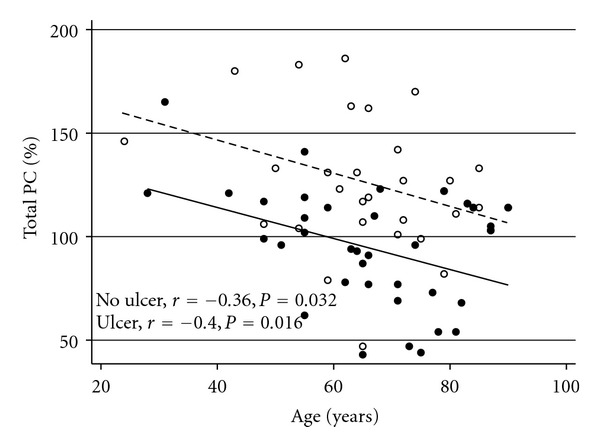
Correlation between total PC and age for patients with no ulcer (open circles, upper dotted line) and patients with lower leg ulcers (closed circles, lower full line).

Of the 72 patients with diabetes in this study, 36 had chronic (>6 month duration) lower leg ulcers, and 36 patients did not have any ulcers (control group). All dependent variables were normally distributed, except for sEPCR and HbA_1c_ which required log transformation. Results are shown in Table 1. Between the 2 groups, there was no difference in age, sex, duration of diabetes, or HbA_1c_. There was no correlation between PC and gender, type of diabetes, HbA_1c_, or CRP. There was a negative correlation between PC levels and age (*r* = −0.38, *P* = 0.03) that remained when groups were analysed separately (Figure 1). The most striking difference was the significantly lower levels of plasma total PC in patients with lower leg ulcers compared to control subjects (*P* < 0.001). Of the 36 patients with lower leg ulcers, 8 had total PC levels that were below the normal range (70%–180%), whereas only 1 of 36 control patients was lower than normal (Fisher's Exact *P* = 0.028). Ulcer type was not a significant covariate for plasma total PC (*P* = 0.35). Levels were 105 ± 26% for neuropathic (*n* = 14) ulcers, 84 ± 29% for ischaemic ulcers (*n* = 10), and 91 ± 29% for mixed neuropathic/ischaemic (*n* = 11). The protein C level of the only patient with a venous ulcer was 116%.

There was no difference in protein S, APTT, or fibrinogen levels between the 2 groups; however, there was significantly higher INR, C reactive protein, and prothrombin time in patients with ulcers. INR was negatively correlated with PC in the ulcer group only (*r* = −0.57, *P* = 0.001). Plasma sEPCR levels did not statistically differ in patients with diabetes who had lower leg ulcers compared with matched controls. 

## 4. Discussion

This is the first study to examine circulating PC levels in patients with diabetes who have lower leg ulcers compared to those without ulcers. Our results show that patients with diabetes who have lower leg ulcers have lower levels of plasma total PC than their counterparts without ulcers. There was no difference in Protein S, APTT, fibrinogen, or sEPCR between the two patient groups, suggesting that the low total PC levels in these patients are not a direct result of an altered coagulation profile. Blood glucose control appears to be unrelated to low PC levels in patients with lower leg ulcers, as there was no difference in HbA_1c_ between the two groups. 

Test results were not available for all patients, and thus patient numbers are reduced for some tests, particularly CRP. Nonetheless, when corrected for age, there was a significant increase in CRP in patients with lower leg ulcers compared to those without ulcers, suggesting increased inflammation, which is a feature of diabetic skin ulceration. A further limitation to this study is that data on other diabetes complications, such as denervation or arteriopathy which may contribute to failed wound healing, were not available and may confound the observed relationships.

Liu et al. [21] have recently shown that high wound fluid concentrations of matrix metalloproteinase-(MMP-9) predict poor wound healing in diabetic foot ulcers. Considering that APC inhibits MMP-9 production by monocytes [17], it would be interesting to determine whether reduced APC in wound fluid results in increased MMP-9. Regardless of the mechanism, our results show an association between low total PC levels and lower leg ulcers in patients with diabetes. Whilst ~20% of patients with diabetes and lower leg ulcers had total PC levels lower than the normal range, the mean total PC level (96%) of this group fell within the broad normal range (70%–180%). This demonstrates that it may not be necessary for total PC levels to be lower than the normal range for lower leg ulcers to occur. Further work is required to delineate what PC level constitutes “low” in terms of failed wound healing in diabetes. 

Treatment of diabetic lower leg ulcers frequently presents a management challenge as they often respond poorly to conventional wound management therapy, often due to complications such as neuropathy and peripheral vascular disease. We found no significant difference between these different ulcer types in this study. Previous evidence indicates that exogenous APC promotes healing of recalcitrant ulcers in patients with diabetes, acting via numerous different mechanisms including inhibition of inflammation, stimulation of angiogenesis, and reepithelialisation [8, 14, 15]. APC is emerging as a potential therapeutic agent not only for lower leg ulcers but also for a number of other disorders including lung injury, spinal cord injury, and kidney injury [9]. Interestingly, plasma total PC levels are substantially decreased in patients who develop severe sepsis, and the level of total PC correlates inversely with morbidity and mortality [22]. After showing positive results in a clinical trial [7] and obtaining FDA approval for APC to treat sepsis in 10 years previously, Eli Lilly, the company who marketed the drug, controversially withdrew APC from the market in 2011. 

Low circulating PC levels may either predict or be a consequence of lower leg ulcers. However, when combined with our previous findings that (i) APC treatment stimulates healing of lower leg ulcers [8, 9] and (ii) total PC expression in skin surrounding lower leg ulcers is low [9], the current findings provide supportive evidence that low PC levels predispose to lower leg ulcers which do not heal in patients with diabetes. It is feasible that a blood test to measure PC may assist clinicians in the difficult judgement of whether a diabetic ulcer will heal or not. Further longitudinal clinical studies will help confirm the value of such a test. 

## Figures and Tables

**Table 1:** Demographics and laboratory data of patients with diabetes with and without lower leg ulcers. *P* values for differences between patients with and without ulcers were performed by linear regression, corrected for age.

	*n*	Normal range	Diabetes without ulcers (mean ± s.d)	Diabetes with ulcers (mean ± s.d)	*P* value by LR (age as covariate)
Age	72	—	59.82 ± 16.31	65.56 ± 15.15	0.46
Gender	72	—	64% male	64% male	—
Type of diabetes	72	—	82% Type 2	83% Type 2	—
Total PC (%)	72	70–180	127.1 ± 34.0	103.3 ± 22.7	<0.001
Free PS (%)	60	60–160	124.7 ± 22.4	108.4 ± 37.8	0.056
Fibrinogen (g/L)	60	5.9–11.8	11.6 ± 2.23	12.3 ± 2.6	0.76
INR	60	0.8–1.2	0.97 ± 0.08	1.02 ± 0.08	0.003
Prothrombin time (sec)	60	13–15	13.33 ± 0.83	13.90 ± 0.82	0.026
APTT (sec)	60	26–36	27.28 ± 2.512	28.75 ± 2.75	0.12
CRP (mg/L)	32	<47	61.0 ± 62.5	211.4 ± 262.0	0.022
HbA_1c_ (%)	54	4–6	6.96 ± 1.42	7.15 ± 1.79	0.61
sEPCR (ng/mL)	54	—	25.0 ± 15.9	28.0 ± 20.1	0.77

## Abbreviations

APC: Activated protein C
APPT: Activated partial thromboplastin time
CRP: C-reactive protein
EPCR: Endothelial protein C receptor
INR: International Normalised Ratio
MMP: Matrix metalloproteinase
PC: Protein C
PT: Prothrombin time
sEPCR: Soluble endothelial protein C receptor.

## References

[1] Esmon CT (2000). The anticoagulant and anti-inflammatory roles of the protein C anticoagulant pathway. *Journal of Autoimmunity*.

[2] Mosnier LO, Zlokovic BV, Griffin JH (2007). The cytoprotective protein C pathway. *Blood*.

[3] Xue M, Minhas N, Chow SO (2010). Endogenous protein C is essential for the functional integrity of human endothelial cells. *Cellular and Molecular Life Sciences*.

[4] Xue M, Chow SO, Dervish S, Chan YK, Julovi S, Jackson CJ (2010). Activated protein C enhances human keratinocyte barrier integrity via sequential activation of epidermal growth factor receptor and tie2. *The Journal of Biological Chemistry*.

[5] Fukudome K, Kurosawa S, Stearns-Kurosawa DJ, He X, Rezaie AR, Esmon CT (1996). The endothelial cell protein C receptor. Cell surface expression and direct ligand binding by the soluble receptor. *The Journal of Biological Chemistry*.

[6] Kurosawa S, Stearns-Kurosawa DJ, Hidari N, Esmon CT (1997). Identification of functional endothelial protein C receptor in human plasma. *Journal of Clinical Investigation*.

[7] Bernard GR, Vincent JL, Laterre PF (2001). Efficacy and safety of recombinant human activated protein C for severe sepsis. *The New England Journal of Medicine*.

[8] Whitmont K, Reid I, Tritton S (2008). Treatment of chronic leg ulcers with topical activated protein C. *Archives of Dermatology*.

[9] Jackson C, Whitmont K, Tritton S, March L, Sambrook P, Xue M (2008). New therapeutic applications for the anticoagulant, activated protein C. *Expert Opinion on Biological Therapy*.

[10] Zlokovic BV, Zhang C, Liu D, Fernandez J, Griffin JH, Chopp M (2005). Functional recovery after embolic stroke in rodents by activated protein C. *Annals of Neurology*.

[11] Isermann B, Vinnikov IA, Madhusudhan T (2007). Activated protein C protects against diabetic nephropathy by inhibiting endothelial and podocyte apoptosis. *Nature Medicine*.

[12] Yasui H, Gabazza EC, Tamaki S (2001). Intratracheal administration of activated protein C inhibits bleomycin-induced lung fibrosis in the mouse. *American Journal of Respiratory and Critical Care Medicine*.

[13] Kumral A, Yesilirmak DC, Tugyan K (2010). Activated protein C reduces intestinal injury in an experimental model of necrotizing enterocolitis. *Journal of Pediatric Surgery*.

[14] Jackson CJ, Xue M, Thompson P (2005). Activated protein C prevents inflammation yet stimulates angiogenesis to promote cutaneous wound healing. *Wound Repair and Regeneration*.

[15] Xue M, Thompson P, Kelso I, Jackson C (2004). Activated protein C stimulates proliferation, migration and wound closure, inhibits apoptosis and upregulates MMP-2 activity in cultured human keratinocytes. *Experimental Cell Research*.

[16] Xue M, Campbell D, Sambrook PN, Fukudome K, Jackson CJ (2005). Endothelial protein C receptor and protease-activated receptor-1 mediate induction of a wound-healing phenotype in human keratinocytes by activated protein C. *Journal of Investigative Dermatology*.

[17] Xue M, Campbell D, Jackson CJ (2007). Protein C is an autocrine growth factor for human skin keratinocytes. *The Journal of Biological Chemistry*.

[18] Expert Committee on the Diagnosis and Classification of Diabetes Mellitus (2003). Report of the expert committee on the diagnosis and classification of diabetes mellitus. *Diabetes Care*.

[19] Vukovich TC, Schernthaner G (1986). Decreased protein C levels in patients with insulin-dependent type I diabetes mellitus. *Diabetes*.

[20] Yano Y, Gabazza EC, Kitagawa N (2004). Tumor necrosis, factor-*α* is associated with increased protein C activation in nonobese type 2 diabetic patients. *Diabetes Care*.

[21] Liu Y, Min D, Bolton T (2009). Increased matrix metalloproteinase-9 predicts poor wound healing in diabetic foot ulcers. *Diabetes Care*.

[22] Esmon CT (2002). Protein C pathway in sepsis. *Annals of Medicine*.

